# Heart Failure in Lebanon: A Glimpse into the Reality of Growing Burden

**DOI:** 10.3390/diagnostics16010057

**Published:** 2025-12-24

**Authors:** Zahraa Saker, Mohamad Hamieh, Fadi Abdel-Sater, Ali Rabah

**Affiliations:** 1 Doctorate School of Science and Technology, Lebanese University, Beirut P.O. Box 6573/14, Lebanon; 2 Department of Cardiology, Beirut Cardiac Institute, Beirut, Lebanon; 3 Department of Biology, Lebanese University, Beirut P.O. Box 6573/14, Lebanon; 4 Department of Cardiac Electrophysiology, Beirut Cardiac Institute, Beirut, Lebanon

**Keywords:** ejection fraction, heart failure, mortality, survival, treatment

## Abstract

**Objective**: This study examined the clinical characteristics, management strategies, and outcomes of heart failure (HF) patients in the Lebanese population to address knowledge gaps regarding comorbidities, adherence to guideline-directed medical therapy, and mortality. **Methods**: The study included 835 patients aged 18 years or older who were hospitalized for HF at a tertiary center between January 2020 and December 2023. Data encompassed demographics, medical history, treatments, and all-cause mortality. **Results**: The median age was 70.0 years (IQR: 62.0–79.0), with males comprising 60.8%. The most common comorbidities were hypertension (84.2%), coronary artery disease (74.6%), diabetes mellitus (55.2%), and atrial fibrillation (33.4%). Males had more ICD (17.1% vs. 8.6%, *p* < 0.001) and CRT-D implants (8.9% vs. 3.7%, *p* = 0.004), and higher coronary artery disease frequency (78.3% vs. 68.8%; *p* = 0.002), whereas females had higher rates of atrial fibrillation (39.4% vs. 29.5%; *p* = 0.003). HF patients with reduced ejection fraction were the most common (56.4%), followed by HF with preserved ejection fraction (30.1%). HF with preserved ejection fraction had the highest rates of hypertension (89.6%) and atrial fibrillation (39.4%). Utilization of ARNI (β = −0.423, HR = 0.655, *p* = 0.041, 95% CI: 0.437–0.983) and SGLT2i (β = −0.432, HR = 0.649, *p* = 0.035, 95% CI: 0.435–0.969) were linked to 34.5% and 35.1% reductions in mortality risk, respectively. **Conclusions**: These results highlight the substantial burden of HF in Lebanon. Distinct demographic and clinical patterns were identified by gender and left ventricular ejection fraction groups. The findings underscore the need for population-specific screening, management strategies, and targeted interventions to improve HF outcomes.

## 1. Introduction

Heart failure (HF) is a complex and progressive medical condition that poses a growing public health challenge, primarily due to its rising prevalence and burden on healthcare systems [1]. Globally, HF affects about 3% of the population, with up to a 24% lifetime risk of developing the condition [2]. The number of cases is expected to increase, driven by aging populations. Notably, HF is particularly common among older adults, with some studies indicating proportions considerably higher than the general prevalence, emphasizing its substantial impact on this age group [3]. This growing burden of HF reduces quality of life and increases healthcare costs, affecting both patients and public health agencies [4]. Currently, cardiovascular diseases (CVD) remain the leading cause of mortality worldwide [5] with HF being a major contributor, accounting for about half of all CVD-related deaths [2].

The most common risk factors that contribute to the development of HF include older age, ischemic heart disease, valvular heart disease, coronary artery disease (CAD), hypertension (HTN), diabetes mellitus (DM), obesity, and gender [1,6]. Other studies have identified genetic variants, smoking, cardiotoxicity with certain drugs, tachycardia, and stress as significant modifiable risk factors [7,8,9,10].

In the Middle East, comprehensive HF studies are limited, despite the observed rise in risk factors associated with HF development. In Lebanon, the worsening of CAD risk factors, combined with unique demographic features—such as an aging population, high urbanization, and lifestyle factors like dietary habits and smoking rates—highlights the need to better understand the clinical profile of HF for effective management and resource distribution. In this article, we aim to provide detailed insights into the clinical profile, demographic characteristics, comorbidities, and mortality rates of patients with HF at a tertiary center in Lebanon, with a particular focus on current treatment strategies.

## 2. Materials and Methods

### 2.1. Study Design, Patient Selection, and Data Collection

A retrospective cohort study was conducted at Beirut Cardiac Institute (BCI), a high-volume tertiary hospital in Lebanon and the Middle East, between 1 January 2020 and 31 December 2023. The current study included 835 adult patients, 18 years or older, with primary hospitalization discharge diagnosis of HF. Duplicate cases, patients with missing echocardiogram results, and files with missing International Classification of Diseases, Tenth Revision (ICD-10) code of HF (ICD-10-I50) were excluded.

Clinical information was collected from the hospital’s archived medical records. Patients’ confidentiality was constantly maintained, and informed consent was waived. Baseline demographic characteristics, medical history, clinical data, laboratory measurements related to HF, and treatment strategies were extracted. Readmission and mortality data were also included. Follow-up was obtained during subsequent readmissions. This study was approved by the Institutional Review Board of Beirut Cardiac Institute, Beirut, Lebanon. Approval Code: 4/2024. Approval Date: 12 January 2024.

### 2.2. Statistical Analysis

Patient characteristics were described as median and interquartile range (IQR) for continuous variables, and as frequencies and percentages for categorical variables. The Pearson Chi-Square test was used to compare categorical variables, and Fisher’s Exact test was used when appropriate. Continuous variables were compared using the Mann–Whitney test and the Kruskal–Wallis H test. All variables with a significant *p*-value were subjected to Bonferroni-adjusted post hoc pairwise comparison. A Kaplan–Meier curve was constructed for the time-to-death, and the log-rank test was applied. A multivariate Cox regression analysis was performed. All statistical analyses were conducted using SPSS v. 24 (SPSS Inc., Chicago, IL, USA). A *p*-value of less than 0.05 was considered statistically significant in all the study.

## 3. Results

**Figure 1:**
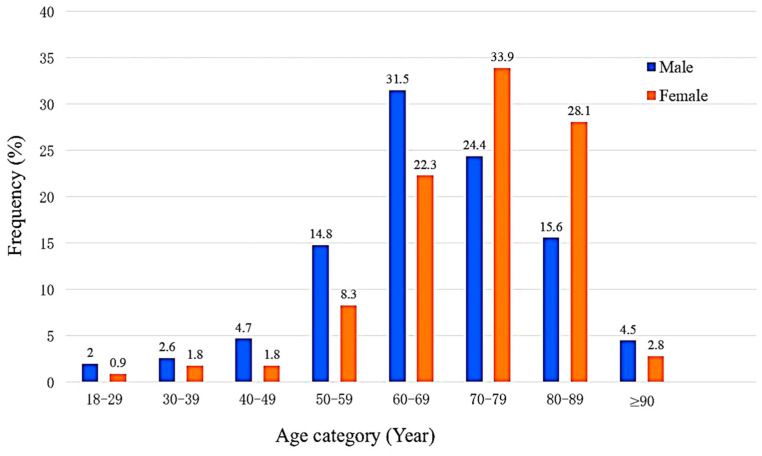
Age-specific distribution of HF patients based on gender.

**Figure 2:**
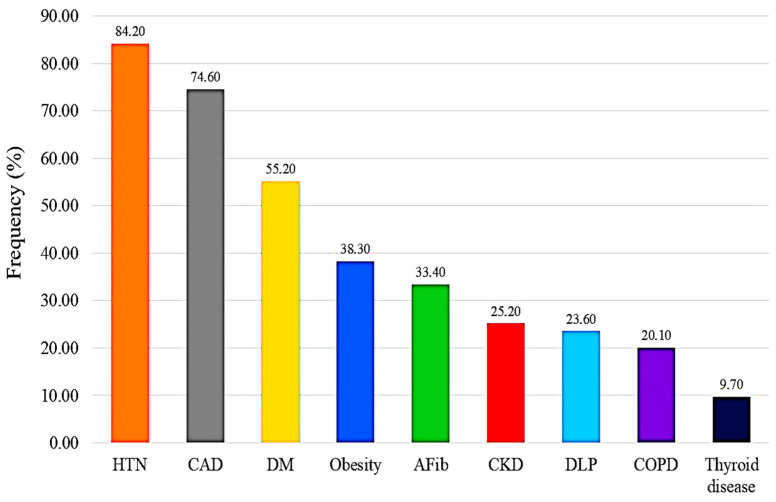
Frequency of the comorbidities in HF patients.

A total of 835 patients with a hospital discharge for HF during 2020–2023 were included in this study, of whom 391 (46.8%) were previously diagnosed with HF. Among those patients, 508 (60.8%) were males with a median age of 70.0 [62.0–79.0] years (Figure 1). Baseline and clinical characteristics are presented in Table 1 and Figure 2. The frequencies of smoking (81.1% vs. 59.0%, *p* < 0.005), ICD (17.1% vs. 8.6%, *p* < 0.001), CRTD (8.9% vs. 3.7%, *p* = 0.004), and CAD (78.3% vs. 68.8%, *p* = 0.002) were higher in males. A significantly greater proportion of females aged ≥70 years (64.8% vs. 44.5%, *p* value < 0.005) and those with atrial fibrillation (AFib) (39.4% vs. 29.5%, *p* = 0.003) than in males (Table 2).

Among the 835 HF patients, 251 (30.1%) had HF with preserved ejection fraction (HFpEF), 113 (13.5%) had HF with mildly reduced EF (HFmrEF), and 471 (56.4%) had HF with reduced EF (HFrEF). The baseline characteristics of HF patients based on left ventricular ejection fraction (LVEF) are detailed in Table 3. HFpEF patients showed the highest rates of HTN (89.6%, *p* = 0.002) and AFib (39.4%, *p* = 0.010), HFrEF patients had the highest frequency of ICD (21.2%, *p* < 0.005) and CRT-D implants (10.6%, *p* < 0.001); whereas, CAD (79.9%, *p* = 0.006) was the most common among patients with HFmrEF.

The prescribed home medical management based on LVEF subgroups is detailed in Table 4. HFpEF patients frequently received class IV antiarrhythmic drugs (AAD) (34.3%, *p* < 0.005) and angiotensin receptor blockers (ARB) (25.5%, *p* < 0.005). A markedly high proportion of HFmrEF patients (94.7%, *p* = 0.015) were managed with beta-blockers. On the other hand, HFrEF patients demonstrated the highest utilization rates of angiotensin receptor-neprilysin inhibitors (ARNI) (27.0%, *p* < 0.005), sodium-glucose co-transporter 2 inhibitors (SGLT2i) (22.5%, *p* < 0.005), class III AAD (25.3%, *p* = 0.020), and angiotensin-converting enzyme inhibitors (ACEi) (39.7%, *p* < 0.005). The use of mineralocorticoid receptor antagonist (MRA), allopurinol, anticoagulants, antiplatelets, diuretics, and statins was comparable across all LVEF-based HF subgroups.

**Figure 3:**
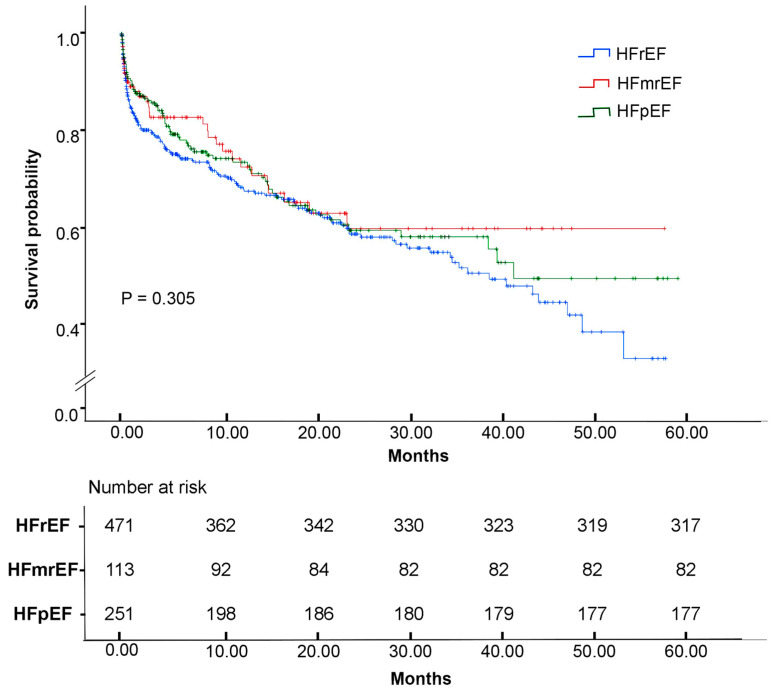
Kaplan–Meier survival analysis based on the left ventricular ejection fraction group.

The median follow-up duration was 2 ± 1 years. All causes of cumulative in-hospital mortality was 259 (31.0%) out of 835 patients. The survival analysis in Figure 3 showed no significant difference in overall survival between HF groups based on LVEF stratification (*p* = 0.305). Additionally, a multivariate Cox proportional hazard regression model was created (Table 5), showing CKD as the strongest independent risk factor, conferring a 72.2% increase in mortality risk (HR = 1.722, *p* < 0.001, 95% CI: 1.278–2.321), followed by EF ≤ 40% which was also significantly associated with a 63.3% increased mortality risk (HR = 1.633, *p* = 0.002, 95% CI: 1.203–2.217), and COPD with a 45.8% increased mortality risk (HR = 1.458, *p* = 0.014, 95% CI: 1.080–1.968). Conversely, treatments with ARNI (β = −0.423, HR = 0.655, *p* = 0.041, 95% CI: 0.437–0.983) and SGLT2i (β = −0.432, HR = 0.649, *p* = 0.035, 95% CI: 0.435–0.969) were linked to a 34.5% and 35.1% reduction in mortality risk, respectively. Other factors, including gender, age ≥ 70 years, smoking, obesity, HTN, DM, CAD, AFib, ACEi use, ARB use, device implants, CRP, and BNP levels, showed no statistically significant association with mortality risk in this analysis.

## 4. Discussion

HF remains a significant global health concern, underscoring the need to understand its clinical characteristics to improve healthcare management and outcomes. Our study provided insights into the clinical profile of HF patients in Lebanon. In this study, the frequency of HF was higher in males than in females, and it increased markedly with age, aligning with the findings from other studies [11,12,13,14]. However, a large-scale cohort study in Turkey [15] and Haiti [16] reported a higher incidence in females compared to males. Although more males were diagnosed with HF compared to females, the proportion of females aged 70 years and older was significantly higher than that of their male counterparts [11,17,18,19].

This study is the first to determine the most common comorbidities among HF patients in Lebanon and the Middle East. HTN (84.2%), CAD (74.6%), DM (55.2%), and AFib (33.4%) were the most frequently observed comorbidities linked to HF. HTN is the leading cause of cardiovascular diseases worldwide, with a prevalence reaching up to 74% [20,21]. Regarding CAD, multiple studies have reported a prevalence of 73% among HF patients [22,23], with a noticeable prevalence in males [24], consistent with our findings. The global prevalence of DM among HF patients ranges from 17% to 47% [15,25], and the high frequencies of overweight (30.5%) and obesity (38.3%) in our study highlight the concomitant increase in DM. On the other hand, AFib prevalence varies widely, with estimates ranging from 10% to 50%, depending on the HF functional class [19,26,27,28] as well as gender disparity, with a higher prevalence in females [29]. These different findings may be attributed to cultural variations and disparities in living standards. Overall, these results position Lebanon among the countries with the highest rates of HF comorbidities.

The prevalence of device implants in HF patients varies depending on the studied population, device type [30], and gender [31]. We observed gender disparities in device implant frequency, with a higher ICD and CRTD use in males, yet females were more likely than males to receive pacemakers. A body of international registries suggested a potential gender difference in the utilization of devices [32,33,34,35,36] suggesting the necessity of controlled research to determine whether this disparity reflects actual clinical differences or indicates systematic biases in referral and implementation practices. Moreover, device implant therapy has been recognized as more likely a primary prevention for patients with HFrEF compared to HFpEF and HFmrEF patients [37,38,39]. Our data regarding gender-related differences in device implantation may reflect a higher frequency of HFrEF in males than in females, or indicate that males derive a greater clinical benefit from ICD or CRT-D implantation.

In the present study, 56.1% of patients hospitalized with HF had a reduced LVEF. Consistent with other research, HFpEF patients were more likely to be older, female, and to have HTN and AFib [39,40,41,42]. However, our study showed a higher frequency of CAD in HFmrEF compared to HFpEF and HFrEF, aligning with other studies [39,41]. An exception was that the three HF phenotypes were similar in the rates of overweight and obesity, DM, dyslipidemia (DLP), chronic kidney disease (CKD), chronic obstructive pulmonary disease (COPD), length of hospitalization, and cumulative in-hospital mortality.

HFrEF patients were treated more frequently with ARNI, SGLT2i, class III AAD, and ACEi [43,44], but less regularly with class IV AAD and ARB than HFpEF and HFmrEF patients. It is worth mentioning that patients with HFmrEF showed a higher rate of beta-blocker use [45]. The therapeutic variability observed in our cohort reflects broader global evidence of demographic underrepresentation and inequitable access to HF therapies. High-risk and older HF patients are frequently undertreated due to demographic, socio-economic, and health-system barriers. Our findings of gender-based differences in device implantation and medication patterns are consistent with these observations [46,47].

In general, HF cumulative mortality remains high, ranging from 15% at 1 year to 75% at 5 years [48] considering that survival rates vary by population and study period. Our reported cumulative in-hospital mortality was comparable to that of other studies, where cumulative in-hospital mortality during the follow-up period did not differ between HFpEF, HFmrEF, and HFrEF [49,50]. Nevertheless, survival differences were observed in other publications [51,52,53]. For instance, a meta-analysis demonstrated that HFrEF patients generally experienced the worst long-term survival outcomes compared to HFmrEF and HFpEF patients [54].

Since the baseline characteristics were significantly different, it is unsurprising that they affect the independent predictors of prognosis, including CKD, COPD, and reduced LVED, consistent with other studies [55,56,57]. A recent cohort study suggested the protective effect of ARNI and SGLT2i among HF groups by maintaining stability and reducing mortality [58]. In contrast to our result, several studies have reported a higher mortality risk in HF males than females [14,59]; whereas others have observed similar mortality risk between genders [60,61]. On the other hand, evidence from randomized controlled trials has shown that monitoring HF patients with implantable devices was associated with lower mortality risk and cardiovascular morbidity [30]. The lack of LVEF stratification for mortality in our analysis likely obscured the significant mortality benefit of ICD in the subgroups of HF patients who meet guideline-based criteria, a finding consistently supported by the recent studies [62].

It is worth noting that the major economic collapse and subsequent hyperinflation in Lebanon caused a severe shortage and unaffordability of essential cardiovascular medications, including ARNI and SGLT2i, forcing patients to neglect their chronic home treatment. This directly led to patients presenting with much more advanced HF. Additionally, economic hardship and COVID-19 restrictions likely raised the threshold for hospitalization, resulting in a pronounced selection bias where only the most severely ill patients sought admission to a major tertiary center. Therefore, our data provide a real-world benchmark demonstrating the devastating combined impact of economic and healthcare system collapse on cardiovascular outcomes.

Our study involved data based on ICD-coded HF diagnoses for administrative purposes. Therefore, some HF patients who were never recorded as hospitalized for HF were not included. Additionally, comorbidities were identified solely from medical records, without the ability to verify the diagnoses. Moreover, we were unable to objectively assess the clinical status to classify patients according to the New York Heart Association classification.

## 5. Conclusions

In conclusion, HF remains a complex medical condition characterized by a wide range of clinical features, necessitating individualized management strategies. Advances in treatment have improved symptoms and quality of life for many patients, yet mortality rates continue to be high, emphasizing the urgency for further therapeutic development. Early diagnosis and comprehensive care are essential in mitigating disease progression and reducing adverse outcomes. Additionally, identifying predictors of worse outcomes may guide the development of new therapeutic strategies. Ongoing research into the pathophysiology of HF will improve our ability to target specific mechanisms and improve prognosis.

## Figures and Tables

**Table 1:** Baseline characteristics of patients hospitalized with heart failure.

Characteristic	*n* = 835 (%)
Previous HF diagnosis	391 (46.8)
Age [IQR] (years)	70.0 [62.0–79.0]
Male	508 (60.8)
Smoking	605 (72.5)
BMI [IQR] (kg/m^2^)	28.7 [24.8–32.6]
Normal weight	188 (22.5)
Overweight	255 (30.5)
Obese	320 (38.3)
Previous cardiac surgery	316 (37.8)
Device implant	208 (24.9)
SBP [IQR] (mmHg)	127.5 [110.0–140.0]
HR [IQR] (bpm)	80.0 [70.0–92.0]
HTN	703 (84.2)
DM	461 (55.2)
DLP	197 (23.6)
CKD	211 (25.2)
Thyroid disease	81 (9.7)
COPD	168 (20.1)
CAD	623 (74.6)
AFib	279 (33.4)
Length of stay [IQR] (days)	6.0 [4.0–9.0]
Hospitalization > 7 days	275 (32.9)
Troponin [IQR] (ng/L)	0.04 [0.02–0.1]
CRP [IQR] (mg/dL)	1.5 [0.7–4.9]
BNP [IQR] (pg/mL)	1038.1 [451.0–2228.1]
TSH [IQR] (µIU/mL)	1.6 [0.7–3.0]
eGFR [IQR] (mL/min/1.73 m^2^)	50.9 [36.5–74.9]
In-hospital mortality	259 (31.0)

Abbreviation: AFib: atrial fibrillation, BMI: body mass index, BNP: brain natriuretic peptide, CAD: coronary artery disease, CKD: chronic kidney disease, COPD: chronic obstructive pulmonary disease, CRP: C-reactive protein, DLP: dyslipidemia, DM: diabetes mellitus, eGFR: estimated glomerular filtration rate, HF: heart failure, HR: heart rate, HTN: hypertension, IQR: interquartile range, SBP: systolic blood pressure, TSH: thyroid stimulating hormone.

**Table 2:** Baseline demographic and clinical characteristics according to gender distribution.

Characteristic	Male *n* = 508 (%)	Female *n* = 327 (%)	*p*-Value
Previous HF diagnosis	233 (45.9)	158 (48.3)	0.488
Age ≥ 70 (years)	226 (44.5)	212 (64.8)	<0.005
Smoking	412 (81.1)	193 (59.0)	<0.005
BMI [IQR] (kg/m^2^)	28.1 [24.8–31.6]	29.1 [25.2–34.2]	0.064
Previous cardiac surgery	193 (38.0)	123 (37.6)	0.913
Device Implant			
ICD	87 (17.1)	28 (8.6)	<0.001
CRT-D	45 (8.9)	12 (3.7)	0.004
Pacemaker	14 (2.8)	22 (6.7)	0.006
HTN	418 (82.3)	285 (87.2)	0.06
DM	270 (53.1)	191 (58.4)	0.136
DLP	117 (23.0)	80 (24.5)	0.634
CKD	131 (25.8)	80 (24.5)	0.668
COPD	94 (18.5)	74 (22.6)	0.147
CAD	398 (78.3)	225 (68.8)	0.002
AFib	150 (29.5)	129 (39.4)	0.003
Length of stay > 7 days	174 (34.3)	101 (30.9)	0.313
One-year re-admission ≥ 2 times due to HF	76 (15.0)	45 (13.8)	0.631
In-hospital mortality	152 (29.9)	107 (32.7)	0.393

Abbreviation: AFib: atrial fibrillation, BMI: body mass index, CAD: coronary artery disease, CKD: chronic kidney disease, COPD: chronic obstructive pulmonary disease, DLP: dyslipidemia, DM: diabetes mellitus, HF: heart failure, HFmrEF: heart failure mildly reduced ejection fraction, HFpEF: heart failure preserved ejection fraction, HFrEF: heart failure reduced ejection fraction, HTN: hypertension, IQR: interquartile range.

**Table 3:** Baseline demographic and clinical characteristics according to LVEF.

Characteristic	HFpEF (EF ≥ 50%) *n* = 251 (%)	HFmrEF (EF 41–49%) *n* = 113 (%)	HFrEF (EF ≤ 40%) *n* = 471 (%)	Overall *p*-Value
De Novo diagnosis	156 (62.2) ^a^	67 (59.3) ^a,b^	221 (46.9) ^b^	<0.005
Male	106 (42.2) ^a^	62 (54.9) ^a^	340 (72.2) ^c^	<0.005
Age ≥ 70	171 (68.1) ^a^	74 (65.5) ^a^	193 (41.0) ^c^	<0.005
Smoking	162 (64.5) ^a^	84 (74.3) ^a,b^	359 (76.2) ^b^	0.003
BMI [IQR] (kg/m^2^)	29.7 [25.5–34.8]	28.7 [24.4–33.7]	28.3 [24.7–31.3]	0.055
Previous cardiac surgery	94 (37.5)	49 (43.4)	173 (36.7)	0.421
Type of implanted device				
ICD	12 (4.8) ^a^	3 (2.7) ^a^	100 (21.2) ^c^	<0.001
CRT-D	4 (1.6) ^a^	3 (2.7) ^a^	50 (10.6) ^c^	<0.001
Pacemaker	18 (7.2) ^b^	3 (2.7) ^a,b^	15 (3.2) ^a^	0.028
HTN	225 (89.6) ^a^	100 (88.5) ^a,b^	378 (80.3) ^b^	0.002
DM	133 (53.0)	67 (59.3)	261 (55.4)	0.539
DLP	59 (23.5)	36 (31.9)	102 (21.7)	0.072
CKD	62 (24.7)	30 (26.5)	119 (25.3)	0.936
COPD	51 (20.3)	28 (24.8)	89 (18.9)	0.383
CAD	169 (67.3) ^c^	90 (79.6) ^a^	364 (77.3) ^a^	0.006
AFib	99 (39.4) ^a^	43 (38.1) ^a,b^	137 (29.1) ^b^	0.010
Length of stay > 7 days	74 (29.5)	37 (32.7)	164 (34.8)	0.351
One-year re-admission ≥ 2 times due to HF	32 (12.7)	17 (15.0)	72 (15.3)	0.661
In-hospital mortality	74 (259)	31 (27.4)	154 (32.7)	0.455

Abbreviation: AFib: atrial fibrillation, BMI: body mass index, CAD: coronary artery disease, CKD: chronic kidney disease, COPD: chronic obstructive pulmonary disease, DLP: dyslipidemia, DM: diabetes mellitus, HF: heart failure, HFmrEF: heart failure mildly reduced ejection fraction, HFpEF: heart failure preserved ejection fraction, HFrEF: heart failure reduced ejection fraction, HTN: hypertension, IQR: interquartile range, LVEF: left ventricular ejection fraction. ^a,b^: Groups in the raw that share a common superscript letter are not statistically different (P_adj_ > 0.05). ^c^: A group is significantly different from both other groups (P_adj_ < 0.05).

**Table 4:** Home medications according to LVEF.

Home Medication	HFpEF (EF ≥ 50%) *n* = 251 (%)	HFmrEF (EF 41–49%) *n* = 113 (%)	HFrEH (EF ≤ 40%) *n* = 471 (%)	Overall *p*-Value
ARNI	17 (6.8) ^a^	13 (11.5) ^a^	127 (27.0) ^c^	<0.005
SGLT2i	27 (10.8) ^a^	11 (9.7) ^a^	106 (22.5) ^c^	<0.005
MRA	91 (36.3)	46 (40.7)	203 (43.1)	0.204
Beta-blocker	211 (84.1) ^a^	107 (94.7) ^b^	416 (88.3) ^a,b^	0.015
Class III AAD	42 (16.7) ^a^	21 (18.6) ^a,b^	119 (25.3) ^b^	0.020
Class IV AAD	86 (34.3) ^a^	36 (31.9) ^a^	69 (20.4) ^c^	<0.005
ACEi	55 (21.9) ^a^	34 (30.1) ^a,b^	187 (39.7) ^b^	<0.005
ARB	64 (25.5) ^a^	28 (24.8) ^a,b^	78 (16.6) ^b^	0.008
Allopurinol	58 (23.1)	21 (18.6)	94 (20.0)	0.509
Anticoagulant	165 (65.7)	76 (67.3)	296 (62.8)	0.580
Antiplatelet	192 (76.5)	92 (81.4)	392 (83.2)	0.089
Diuretic	213 (84.9)	100 (88.5)	424 (90.0)	0.121
Statin	173 (68.9)	87 (77.0)	356 (75.6)	0.108

Abbreviation: AAD: antiarrhythmic drugs, ACEi: angiotensin-converting enzyme inhibitor, ARB: angiotensin receptor blocker, ARNI: angiotensin receptor-neprilysin inhibitor, LVEF: left ventricular ejection fraction, MRA: mineralocorticoid receptor antagonist, SGLT2i: sodium-glucose co-transporter 2 inhibitor. ^a,b^: Groups in the raw that share a common superscript letter are not statistically different (P_adj_ > 0.05). ^c^: A group is significantly different from both other groups (P_adj_ < 0.05).

**Table 5:** Cox proportional hazard multiple regression analysis for mortality.

Variable	β	*p*-Value	HR	95% CI
Gender	0.183	0.237	1.200	0.887–1.624
Age ≥ 70 years	0.265	0.079	1.304	0.969–1.753
Smoking	0.256	0.137	1.292	0.922–1.812
Obesity	−0.158	0.264	0.854	0.646–1.127
HTN	−0.301	0.142	0.740	0.495–1.106
DM	0.093	0.522	1.097	0.826–1.457
CKD	0.544	<0.001	1.722	1.278–2.321
COPD	0.377	0.014	1.458	1.080–1.968
CAD	0.041	0.819	1.042	0.732–1.484
AFib	0.201	0.155	1.222	0.927–1.613
ARNI	−0.423	0.041	0.655	0.437–0.983
SGLT2i	−0.432	0.035	0.649	0.435–0.969
ACEi	−0.103	0.493	0.902	0.672–1.211
ARB	−0.161	0.362	0.851	0.602–1.204
EF ≤ 40%	0.490	0.002	1.633	1.203–2.217
ICD	−0.274	0.199	0.761	0.501–1.155
CRT-D	−0.083	0.765	0.921	0.535–1.583
Pacemaker	−0.013	0.966	0.987	0.543–1.796
CRP (mg/dL)	0.326	0.056	1.385	0.991–1.935
BNP (pg/mL)	0.243	0.281	1.275	0.820–1.985

Abbreviation: ACEi: angiotensin-converting enzyme inhibitor, AFib: atrial fibrillation, ARB: angiotensin receptor blocker, ARNI: angiotensin receptor-neprilysin inhibitor, β: log hazard ratio, BNP: brain natriuretic peptide, CAD: coronary artery disease, CI: confidence interval, CKD: chronic kidney disease, COPD: chronic obstructive pulmonary disease, CRP: C-reactive protein, DM: diabetes mellitus, EF: ejection fraction, HR: hazard ratio, HTN: hypertension, SGLT2i: sodium-glucose cotransporter-2 inhibitor.

## Data Availability

Data is contained within the article, and further inquiries can be directed to the corresponding author.

## Abbreviations

The following abbreviations are used in this manuscript:

AAD	Antiarrhythmic drugs
ACEi	Angiotensin converting enzyme inhibitor
AFib	Atrial fibrillation
ARB	Angiotensin receptor blocker
ARNI	Angiotensin receptor neprilysin inhibitor
BMI	Body mass index
BNP	Brain natriuretic peptide
CAD	Coronary artery disease
CKD	Chronic kidney disease
COPD	Chronic obstructive pulmonary disease
CRP	C-reactive protein
DM	Diabetes mellitus
EF	Ejection fraction
eGFR	Estimated glomerular filtration rate
HF	Heart failure
HFmrEF	Heart failure with mildly reduced ejection fraction
HFpEF	Heart failure with preserved ejection fraction
HFrEF	Heart failure with reduced ejection fraction
HR	Heart rate
HTN	Hypertension
MRA	Mineralocorticoid receptor antagonist
TSH	Thyroid-stimulating hormone
SGLT2i	Sodium-glucose co-transporter 2 inhibitor
